# Intracranial ependymoma grade II with lipomatous metaplasia: a case report and literature review

**DOI:** 10.3389/fonc.2026.1674547

**Published:** 2026-04-01

**Authors:** Qingbo Wang, Qingze Zeng, Kaicheng Li, Qiaojun Chen, Xiao Luo

**Affiliations:** 1 Department of Radiology, The Second Affiliated Hospital of Zhejiang University School of Medicine, Hangzhou, China; 2 Department of Pathology, The Second Affiliated Hospital of Zhejiang University School of Medicine, Hangzhou, China

**Keywords:** brain mass, ependymoma, lipomatous metaplasia, MRI, oncology

## Abstract

Ependymomas are uncommon tumors arising from ependymal cells of the central nervous system, with lipomatous metaplasia representing an extremely rare pathological variant. We report a case of a 28-year-old male who presented with a 5-year history of an incidentally discovered intracranial mass and recent-onset dizziness. Magnetic resonance imaging (MRI) revealed a left frontotemporal lesion characterized by significant fat signal, progressive enhancement, and mass effect on adjacent structures. The patient underwent gross-total resection, and histopathology confirmed a World Health Organization (WHO) Grade II ependymoma with lipomatous metaplasia. Immunohistochemical analysis further supported the diagnosis, highlighting features of both ependymal differentiation and fatty metaplasia. We also provide a comprehensive literature review of previously reported cases, emphasizing the rarity, imaging characteristics, differential diagnosis, and surgical considerations for this unique pathological variant. This case underscores the importance of careful radiological and pathological evaluation to guide diagnosis and management, and aims to raise awareness of this rare subtype among clinicians and pathologists.

## Introduction

Ependymomas are primary tumors originating from ependymal cells of the central nervous system, accounting for 2%-9% of intracranial tumors and are particularly common in children and adolescents (1, 2). According to the 2021 World Health Organization (WHO) classification of central nervous system tumors, ependymomas are categorized based on anatomic location and molecular features into several subtypes, including supratentorial ependymoma, ZFTA fusion-positive; supratentorial ependymoma, YAP1 fusion-positive; posterior fossa ependymoma, PFA (Group A); posterior fossa ependymoma, PFB (Group B); spinal ependymoma; spinal ependymoma, MYCN-amplified; myxopapillary ependymoma (WHO grade 2); and subependymoma (WHO grade 1), with additional designations like NEC (not elsewhere classified) or NOS (not otherwise specified) for tumors that do not fully match defined criteria (3–6). This classification highlights key distinctions among spinal, supratentorial, and infratentorial (posterior fossa) ependymomas. Supratentorial ependymomas primarily occur in the cerebral hemispheres, often in children and young adults, with ZFTA fusion-positive subtypes associated with poorer prognosis (5-year progression-free survival ~29%) due to aggressive behavior, while YAP1 fusion-positive ones have better outcomes (>90% overall survival). Infratentorial ependymomas, located in the posterior fossa such as the fourth ventricle or cerebellum, show age-related differences: PFA (Group A) predominantly affects young children with dismal prognosis (10-year overall survival ~56%) and high recurrence rates, whereas PFB (Group B) occurs in adolescents and adults with favorable outcomes (10-year overall survival ~88%). Spinal ependymomas are more common in adults, typically in the cervical or thoracic cord, with classic subtypes linked to NF2 mutations and excellent prognosis (>90% overall survival), though MYCN-amplified variants are aggressive with higher dissemination risk. These distinctions influence clinical management, with molecular profiling guiding therapy; for example, historical variants like tanycytic ependymoma are no longer distinct and are reclassified by location and genetics, as seen in a 2023 case of a calcified supratentorial ependymoma mimicking pineal tumors (7). Ependymomas corresponding to WHO grade 2 (e.g., many spinal and myxopapillary subtypes) are among the most common, comprising 50%-60% of cases, characterized by slow growth but local invasiveness, with prognosis influenced by tumor location, extent of resection, and molecular subtype (3–6).

Lipomatous metaplasia, a rare pathological variant, refers to the presence of adipocytes or adipose-like tissue within the tumor, typically considered a result of degenerative changes or aberrant differentiation, often manifesting as intracytoplasmic fat vacuoles that coalesce into large droplets, pushing nuclei to the periphery and creating a signet-ring cell-like appearance (8). In central nervous system tumors, lipomatous metaplasia is more commonly observed in teratomas, lipomas, or gangliogliomas, but its occurrence in ependymomas is rare yet increasingly reported, with over 15 documented cases since its initial descriptions (9–11). Literature on ependymomas with lipomatous metaplasia indicates predominance in children and young adults (ages 2–48 years), with diverse locations across supratentorial, infratentorial, and spinal regions; histological confirmation often requires GFAP positivity in cytoplasmic rims, EMA punctate staining, and ultrastructural evidence of ependymal features like microlumina and osmiophilic fat. Early reports highlighted diagnostic challenges with mimics like metastatic adenocarcinoma, and recent cases incorporate molecular data, such as ZFTA-RELA fusions in supratentorial subtypes (9–16). Approximately half of cases show recurrence, emphasizing the need for complete resection and follow-up. **Table 1** summarizes key reported cases from the literature.

**Table 1:** Summary of key reported cases of ependymoma with lipomatous metaplasia in the literature.

No.	Author (year)	Age/sex	Location	Histological features	Molecular features	Outcome
1	Hirato et al. (1997) (12)	2/Unknown	Left occipital lobe (supratentorial)	Extensive vacuolization, signet-ring cells, perivascular pseudorosettes, GFAP+, EMA+	Not reported	Not reported
2	Ruchoux et al. (1998) (11)	13/F; 16/M; 48/F	Third ventricle; paraventricular hemispheric white matter; hemispheric white matter (supratentorial)	Lipomatous differentiation, fat droplets, GFAP+ in cytoplasmic rims, ependymal rosettes	Not reported	Not reported
3	Sharma et al. (2000) (13)	4/M; 5/M; 9/M; 25/M; 45/M	Supratentorial (4 cases); spinal (1 case)	Extensive lipidization mimicking adipose tissue, GFAP+, no atypia	Not reported	No recurrence (follow-up 6 months to 5 years)
4	Rios et al. (2001) (10)	5/M	Supratentorial (intraventricular)	Lipomatous with fat vacuoles, calcification	Not reported	Not reported
5	Gaur et al. (2016) (9)	13/F	Intraventricular (supratentorial)	Focal perivascular rosettes, adipocytic cells, GFAP+, S100+, vimentin+	Not reported	Recurrence after 1 year
6	Salazar et al. (2017) (14)	16/M	Left paraventricular (supratentorial)	Extensively vacuolated, signet-ring appearance, dystrophic calcifications, GFAP+, EMA+	Not reported	Not reported
7	Li et al. (2025) (15)	15/M	Right parieto-occipital cortex (supratentorial)	Perivascular pseudorosettes, adipose differentiation, GFAP+, vimentin+, S100+, EMA+	ZFTA-RELA fusion	No recurrence at 36 months
8	Rodriguez et al. (2022) (16)	Adult (not specified)	Posterior fossa (fourth ventricle, extending to cerebellum)	Extensive vacuolization, signet-ring cells, perivascular pseudorosettes	Not reported	Not reported

The imaging features of ependymomas typically include iso- or hypointense signals on T1-weighted MRI, hyperintense signals on T2-weighted MRI, and homogeneous or heterogeneous enhancement after contrast administration. In cases with lipomatous metaplasia, hyperintense signals on both T1- and T2-weighted images, along with signal suppression on fat-saturation sequences, may be observed, potentially mimicking fat-containing tumors such as teratomas or cholesterolomas and complicating diagnosis (17). Here, we report a rare case of a 28-year-old male with a left frontotemporal grade II ependymoma exhibiting lipomatous metaplasia. By analyzing its imaging features, clinical course, and pathological findings, this study highlights the diagnostic challenges and clinical significance of this uncommon tumor variant.

## Case presentation

A 28-year-old male presented to our institution in August 2024 with a 3-day history of dizziness. Five years earlier, during a routine physical examination in 2019, an incidental intracranial mass had been identified on brain MRI. At that time, the patient was asymptomatic, and the lesion was small (approximately 20 mm × 25 mm × 30 mm), located in the left temporal lobe, with heterogeneous signals—iso- to hypo-intense on T1-weighted imaging and hyperintense on T2-weighted imaging—showing minimal heterogeneous enhancement after contrast administration. No significant perilesional edema or mass effect was noted, and the findings were suggestive of a low-grade glial tumor or benign lesion, but the patient declined further evaluation or intervention. He remained without symptoms for the subsequent years, with no reported headaches, seizures, visual changes, or neurological deficits. His medical history was unremarkable, with no hypertension, diabetes, prior surgeries, or drug allergies. He had a 5-year smoking history (10 cigarettes per day) but denied alcohol consumption. Family history was negative for neurological or oncologic conditions. On admission, physical examination revealed an alert and oriented individual with equal, round, and reactive pupils, grade V muscle strength in all extremities, negative bilateral Babinski signs, and normal cardiopulmonary and abdominal findings.

**Figure 1:**
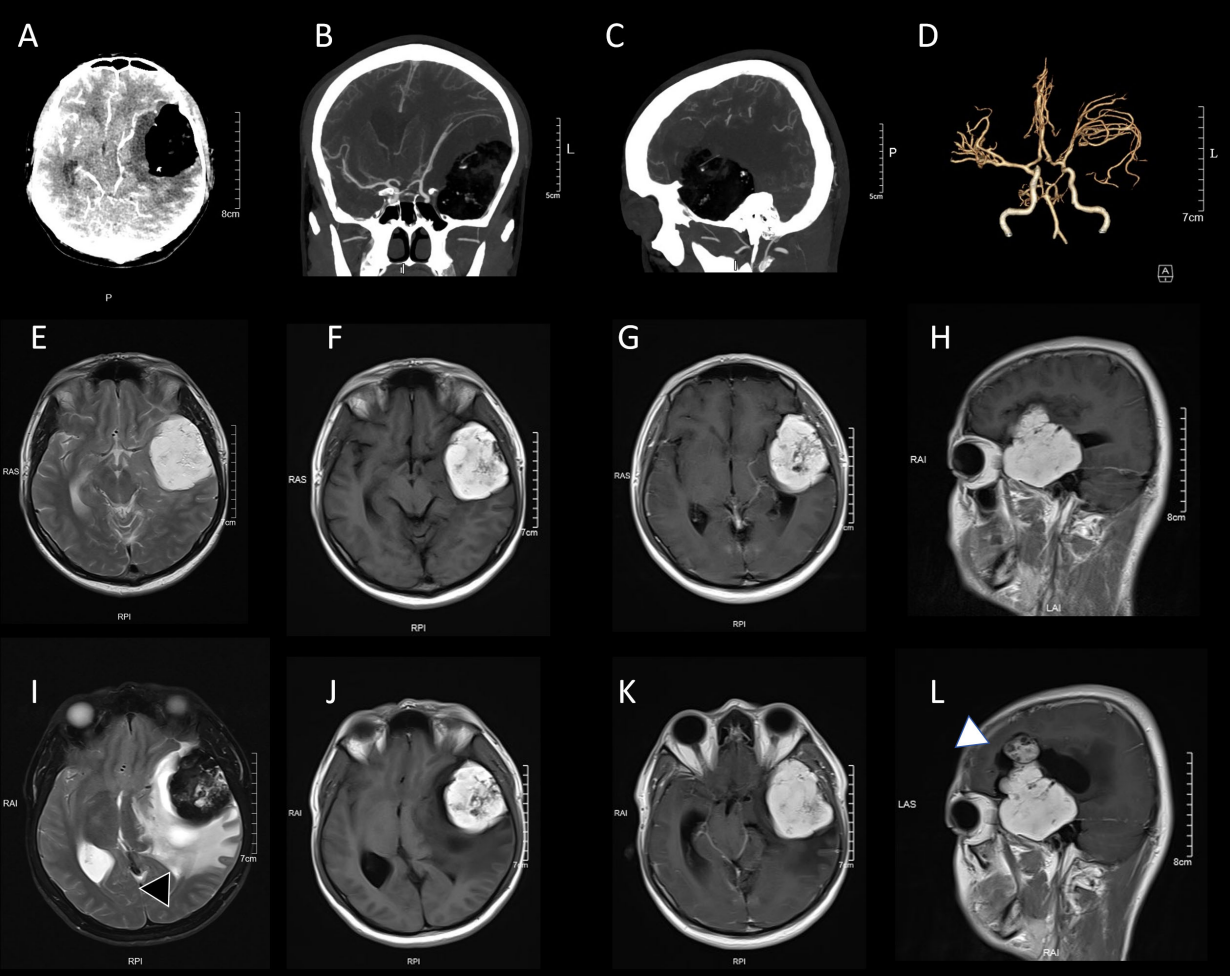
Imaging findings of the case. **(A–D)** Baseline computed tomography angiography (CTA) of the brain revealing a left frontotemporal mass with superior displacement and compression of the left common carotid artery. Images include axial view **(A)**, maximum intensity projection (MIP) coronal view **(B)**, MIP sagittal view **(C)**, and 3D vascular reconstruction **(D)**. **(E–H)** Baseline contrast-enhanced brain magnetic resonance imaging (MRI) demonstrating a large left temporal mass with heterogeneous short T1 and long T2 signals, measuring approximately 47 mm × 59 mm × 72 mm. Fat suppression shows low signal intensity, suggesting high fat content. Post-contrast imaging reveals patchy enhancement within the lesion and nodular enhancement at the superior margin, with compression of surrounding tissues. Images include T2-weighted axial view **(E)**, T1-weighted axial view **(F)**, T1-weighted post-contrast axial view **(G)**, and T1-weighted post-contrast sagittal view **(H)**. **(I–L)** Follow-up contrast-enhanced MRI performed one year later shows persistence of a large left temporal mass with heterogeneous short-T1 and long-T2 signals. Compared with baseline, the enhancement has become more prominent (white arrows), with progression of perilesional edema and mass effect (black arrows). Images include T2-weighted axial **(I)**, T1-weighted axial **(J)**, T1-weighted post-contrast axial **(K)**, and T1-weighted post-contrast sagittal **(L)** views.

Follow-up imaging was not performed until August 2023, when the patient underwent contrast-enhanced brain MRI due to mild intermittent headaches that had developed over the preceding months. The lesion had grown to approximately 47 mm × 59 mm × 72 mm, exhibiting complex signals with short T1 and mixed long T2 components, and low signal intensity on fat-suppression sequences, indicating a prominent fat component. Patchy enhancement was observed post-contrast, along with small nodular hypervascular foci at the superior margin. Mild surrounding edema was present, causing compression of the left lateral ventricle and a slight midline shift, without evidence of herniation. These imaging characteristics raised suspicion for an extra-axial lesion, primarily a teratoma, with cholesterol granuloma as a differential consideration (**Figure 1**). No intervention was pursued at that time, as the patient remained largely asymptomatic aside from the headaches, which were managed conservatively.

In August 2024, following the onset of dizziness, repeat contrast-enhanced MRI demonstrated slight enlargement of the lesion, now extending into the left frontotemporal region and measuring approximately 50 mm × 62 mm × 75 mm. The lesion showed stratified signals, with the upper portion displaying long T1 and T2 signals and the lower portion short T1 and mixed long T2 signals. Fat components were confirmed by signal suppression on fat-saturated sequences, and enhancement had become more extensive and nodular, particularly at the superior margin. Perilesional edema had progressed to extensive and patchy involvement, resulting in a more pronounced mass effect and rightward midline shift. Although the overall size increase was minimal compared to 2023, the enhanced enhancement and worsening edema suggested possible changes in tumor biology, potentially associated with lipomatous metaplasia. These findings prompted surgical intervention.

The patient underwent resection of the left deep cerebral lesion in August 2024, utilizing intraoperative neuroendoscopic assistance to enhance visualization and ensure complete removal. Under general anesthesia, a left frontotemporal craniotomy was performed, providing access to the temporal lobe where the tumor was located. The lesion appeared yellowish and fat-like grossly, with focal calcifications, a firm texture, moderate vascularity, and well-defined margins. Microscopic dissection allowed piecemeal excision of the mixed fatty and solid components, with careful removal of calcified areas to avoid damage to surrounding brain tissue. Endoscopic assistance was employed to inspect the resection cavity, particularly in deep or obscured regions, revealing a small residual nodule at the superior margin that was not visible under direct microscopy; this was subsequently fully excised using the endoscope for guidance and precision. Intraoperative images captured the tumor’s gross appearance and the endoscopic view of the residual nodule prior to its removal (Figure 3). Hemostasis was achieved without complications, with an estimated blood loss of 200 mL and no need for transfusion.

**Figure 2:**
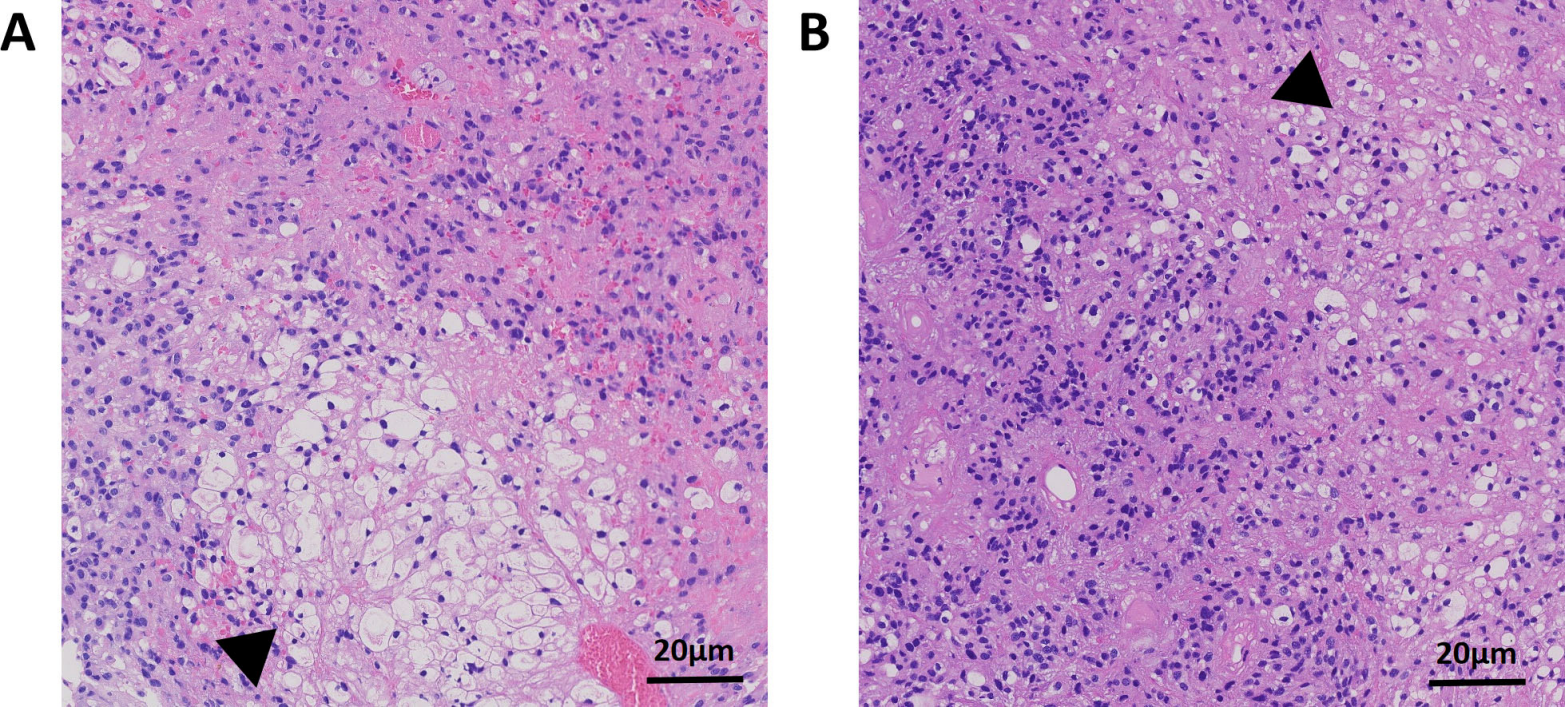
Histopathological findings. **(A)** Histopathological examination of the resected specimen (August 2024) reveals a moderately cellular glial neoplasm composed of atypical ependymal cells forming characteristic perivascular pseudorosettes. Mitotic figures are rare, and no necrosis or microvascular proliferation is identified, consistent with a CNS WHO grade 2 ependymoma. Scattered clusters of mature adipocytes are present within the tumor, indicating lipomatous metaplasia. **(B)** A separate H&E-stained section from the same lesion demonstrates prominent areas of lipomatous metaplasia, with well-differentiated adipocytes embedded within the ependymal tumor tissue. Black arrows indicate areas of lipomatous metaplasia.

Histopathological examination of the resected specimen, conducted in August 2024, demonstrated proliferation of atypical glial cells forming perivascular pseudorosettes, with rare mitotic figures, consistent with a WHO Grade II ependymoma. Clusters of mature adipocytes were interspersed within the tumor, confirming the rare variant of WHO Grade II ependymoma with lipomatous metaplasia (**Figure 2**). Histopathological examination of the left cerebral hemisphere lesion was consistent with a supratentorial ependymoma, NOS, CNS WHO grade 2, with prominent areas of lipomatous metaplasia. Immunohistochemistry showed GFAP(+), Vimentin(+), partial positivity for S100, Nestin, Synaptophysin, and MAP-2, and focal perinuclear dot-like positivity for EMA. Olig2, NeuN, Chromogranin A, IDH1 (R132H), BRAF V600E, SOX10, CK-pan, HMB45, STAT6, and SSTR2 were negative. ATRX expression was retained, p53 staining was low (5–10%), H3 K27M was negative, and H3 K27me3 expression was preserved. The Ki-67 proliferation index was approximately 5%. Mismatch repair proteins (MLH1, MSH2, MSH6, and PMS2) and INI1/SMARCB1 were preserved. Molecular testing was performed using Sanger sequencing on FFPE tissue with adequate tumor content (≈80%). IDH2 exon 4 was wild type, and no hotspot mutations were detected. IDH1 exon 4 testing was ordered but remained pending at the time of reporting. The assay had a detection threshold of ~20% mutant allele frequency, and mutations in other exons could not be excluded. Postoperative CT imaging showed resolution of any hematoma, effusion, or pneumocephalus, with no residual lesion, marked reduction in edema, and correction of the midline shift, without signs of herniation.

During the hospital course, the patient received mannitol for intracranial pressure management, prophylactic antibiotics (cefazolin and linezolid), and supportive intravenous fluids and nutrition. On postoperative day 5, laboratory evaluation revealed mild hypokalemia (3.35 mmol/L) and hypoalbuminemia (31.8 g/L), which were addressed with electrolyte supplementation and enhanced nutritional support. The dizziness resolved promptly, and recovery proceeded without further complications. The patient was discharged after a 10-day stay in August 2024, with recommendations for rehabilitation and a one-month follow-up. At the November 2024 outpatient visit, he reported no pain and was in good general condition, though he had experienced significant weight loss (height: 175 cm, weight: 54 kg, BMI: 17.63). The presumed cause was postoperative cachexia related to reduced appetite and metabolic stress from surgery and recovery; further evaluation by the nutrition department, including dietary assessment and blood work to rule out malabsorption or underlying malignancy, confirmed no additional pathology, and a nutritional plan was initiated to address this. To illustrate the chronological progression of the case, **Table 2** summarizes key events and imaging findings.

**Table 2:** Chronological summary of clinical course and serial imaging findings.

Date	Event/imaging findings
2019	Incidental detection of small left temporal lesion (20 mm × 25 mm × 30 mm) on MRI: heterogeneous signals, minimal enhancement, no edema or mass effect. Asymptomatic; no intervention.
2021	No imaging performed; patient remained asymptomatic.
August 2023	MRI shows growth to 47 mm × 59 mm × 72 mm: complex signals with fat component, patchy enhancement, mild edema, slight midline shift. Mild headaches noted; conservative management.
August 2024	MRI reveals slight enlargement to 50 mm × 62 mm × 75 mm: stratified signals, extensive nodular enhancement, worsened edema, pronounced midline shift. Dizziness onset; surgical resection performed.
August 2024 (post-op)	CT confirms complete resection, resolution of edema and shift. Histopathology: Grade II ependymoma with lipomatous metaplasia.
November 2024	Follow-up: No recurrence on imaging; weight loss noted, attributed to postoperative cachexia; nutritional evaluation and plan initiated.

## Discussion

Ependymomas with lipomatous metaplasia represent a rare variant characterized by the presence of adipocytes or adipose-like tissue within the tumor, often resulting from degenerative changes or aberrant differentiation. This phenomenon was first described in 1997 by Hirato et al. in a 2-year-old patient with a supratentorial occipital lesion exhibiting extensive vacuolization and signet-ring cells (12). Since then, approximately 10 cases have been reported, predominantly in children and adolescents, with a slight male predominance noted in series such as Sharma et al.’s 2000 report of five males (13). These tumors can occur in various locations, including supratentorial (e.g., intraventricular, paraventricular, or cortical), infratentorial (posterior fossa, such as the fourth ventricle with cerebellar extension), and spinal (rarely, in the thoracolumbar region), and are typically graded WHO 2-3. Histologically, the metaplasia manifests as intracytoplasmic fat vacuoles that coalesce into signet-ring appearances or, less commonly, as mature adipocytes, as seen in the present case. Immunohistochemistry (IHC) usually reveals GFAP positivity in cytoplasmic rims, EMA dot-like perinuclear staining, vimentin and S100 positivity, and low Ki-67 proliferation indices (<5-10%), which help differentiate it from metastatic adenocarcinoma, liponeurocytoma, or xanthomatous changes. Ultrastructurally, microlumina with microvilli and osmiophilic fat confirm the ependymal origin. Radiologically, these tumors show hyperintense signals on T1- and T2-weighted imaging that suppress on fat-saturation sequences, along with heterogeneous enhancement and calcifications, potentially mimicking teratomas or lipomas. Prognosis varies, with approximately 50% recurrence rates emphasizing the importance of gross total resection; 5-year progression-free survival is about 70-90% for grade 2 tumors but lower in cases with anaplastic features.

This case report details a rare WHO Grade II ependymoma with lipomatous metaplasia in the left frontotemporal region of a 28-year-old male, posing diagnostic challenges due to its atypical imaging and pathological features. The initial MRI in 2019 identified a small lesion with heterogeneous signals and minimal enhancement, which progressed slowly over five years. By 2023, the lesion displayed complex signals with short T1 and mixed long T2 components, low signal on fat-suppression sequences indicating substantial fatty content, and patchy nodular enhancement suggesting active vascularity (1). A follow-up MRI in 2024 revealed increased enhancement, worsened edema, and progressive mass effect, despite minimal change in size. In contrast to typical ependymomas—which exhibit iso- or hypointense T1 signals, hyperintense T2 signals, and homogeneous enhancement—these features resembled those of fat-containing tumors such as teratomas, cholesterol granulomas, or lipomas, leading to a preoperative diagnosis of teratoma (17, 18). Postoperative pathology confirmed WHO Grade II ependymoma with lipomatous metaplasia, featuring perivascular pseudorosettes and clusters of mature adipocytes interspersed within the tumor.

Unlike most reported ependymomas with lipomatous metaplasia, which primarily show lipid accumulation as vacuole formation resembling signet-ring cells, this case demonstrated true mature adipocytes, suggesting a distinct pathological mechanism possibly related to long-standing tumor degeneration or aberrant differentiation (10, 11, 14).For instance, a prior case involving a left frontal ependymoma in a 16-year-old male described signet-ring-like cells and lipid vacuoles, while a fourth ventricle ependymoma attributed lipomatous metaplasia to tumor heterogeneity or metabolic abnormalities (19). The underlying mechanism of lipomatous metaplasia remains poorly understood but may involve tumor cell pluripotency, ectopic differentiation, or microenvironmental changes (8). Molecular analyses, such as whole-exome sequencing or epigenetic profiling, could elucidate associations with ependymoma subtypes (e.g., RELA or YAP1 fusions) and inform precision treatments (20).

The imaging findings required differentiation from other intracranial tumors with fatty or enhancing characteristics. Teratomas, often midline, display short T1 and high T2 signals with calcifications and heterogeneous enhancement but typically include ectodermal components like hair, which were absent in this frontotemporal lesion (21). Cholesterol granulomas, common in the skull base, lack significant enhancement, contrasting with the prominent enhancement observed here. Intracranial lipomas, as congenital lesions, show uniform short T1 and high T2 signals without enhancement or edema, making them unlikely (22). Meningiomas, gliomas, gangliogliomas, and degenerative schwannomas were ruled out due to the absence of dural tail signs, astrocytic proliferation, epilepsy, or nerve pathway involvement, respectively.

Literature on ependymomas with lipomatous metaplasia is sparse, encompassing a few cases across Grades II and III, including a 13-year-old female with an intraventricular ependymoma that recurred one year post-surgery (9). This supratentorial Grade II ependymoma, managed with gross total resection, exhibited progressive behavior over five years without intervention, with increased enhancement and edema indicating heightened biological activity potentially linked to lipomatous metaplasia. Although Grade II ependymomas generally have a favorable prognosis, the progression observed and reported recurrences in about half of similar cases suggest that lipomatous metaplasia may influence tumor behavior or reflect underlying heterogeneity (14). Recent molecular classifications, such as RELA fusions in supratentorial ependymomas, delineate distinct subtypes, yet the molecular basis of lipomatous metaplasia remains unclear due to the paucity of cases (4). Future studies employing RNA sequencing or metabolomics could uncover its drivers and facilitate targeted therapies.

## Conclusion

This case highlights the diagnostic complexity of WHO Grade II ependymoma with lipomatous metaplasia, stemming from its imaging similarities to fat-containing tumors and distinctive pathology. Histological confirmation proved essential for accurate diagnosis, underscoring the value of thorough pathological evaluation. The potential link between lipomatous metaplasia and progressive behavior, even in low-grade ependymomas, calls for vigilant long-term imaging surveillance and prompt intervention. This report adds valuable insights to this rare variant, advocating for molecular studies to deepen understanding and refine management strategies.

## Data Availability

The raw data supporting the conclusions of this article will be made available by the authors, without undue reservation.

## References

[1] Louis DN Perry A Wesseling P Brat DJ Cree IA Figarella-Branger D . The 2021 WHO classification of tumors of the central nervous system: a summary. Neuro Oncol. (2021) 23:1231–51. doi: 10.1093/neuonc/noab106, PMID: 34185076 PMC8328013

[2] Wu J Armstrong TS Gilbert MR . Biology and management of ependymomas. Neuro Oncol. (2016) 18:902–13. doi: 10.1093/neuonc/now016, PMID: 27022130 PMC4896548

[3] Bertero L Ricci AA Tampieri C Cassoni P Modena P . Ependymomas. Pathologica. (2022) 114:436–46. doi: 10.32074/1591-951X-817, PMID: 36534422 PMC9763977

[4] Pajtler KW Witt H Sill M Jones DT Hovestadt V Kratochwil F . Molecular classification of ependymal tumors across all CNS compartments, histopathological grades, and age groups. Cancer Cell. (2015) 27:728–43. 10.1016/j.ccell.2015.04.002 PMC4712639 25965575

[5] Kresbach C Neyazi S Schuller U . Updates in the classification of ependymal neoplasms: The 2021 WHO Classification and beyond. Brain Pathol. (2022) 32:e13068. doi: 10.1111/bpa.13068, PMID: 35307892 PMC9245931

[6] Mu W Dahmoush H . Classification and neuroimaging of ependymal tumors. Front Pediatr. (2023) 11:1181211. doi: 10.3389/fped.2023.1181211, PMID: 37287627 PMC10242666

[7] Yangi K Yavuz AY Percinoglu G Aki B Celik SE . A huge calcified supratentorial ependymoma: A case report. Cureus. (2023) 15:e37493. doi: 10.7759/cureus.37493, PMID: 37064720 PMC10096750

[8] Lehman NL . Central nervous system tumors with ependymal features: a broadened spectrum of primarily ependymal differentiation? J Neuropathol Exp Neurol. (2008) 67:177–88. doi: 10.1097/NEN.0b013e31816543a6, PMID: 18344909

[9] Gaur K Batra VV Gupta R Sharma MC Narang P Pandey PN . Lipomatous ependymoma: report of a rare differentiation pattern with a comprehensive review of literature. Brain Tumor Pathol. (2016) 33:209–15. doi: 10.1007/s10014-016-0253-9, PMID: 26942599

[10] Chang WE Finn LS . MR appearance of lipomatous ependymoma in a 5-year-old boy. AJR Am J Roentgenol. (2001) 177:1475–8. doi: 10.2214/ajr.177.6.1771475, PMID: 11717110

[11] Ruchoux MM Kepes JJ Dhellemmes P Hamon M Maurage CA Lecomte M . Lipomatous differentiation in ependymomas: a report of three cases and comparison with similar changes reported in other central nervous system neoplasms of neuroectodermal origin. Am J Surg Pathol. (1998) 22:338–46. doi: 10.1097/00000478-199803000-00009, PMID: 9500776

[12] Hirato J Nakazato Y Iijima M Yokoo H Sasaki A Yokota M . An unusual variant of ependymoma with extensive tumor cell vacuolization. Acta Neuropathol. (1997) 93:310–6. doi: 10.1007/s004010050620, PMID: 9083565

[13] Sharma MC Arora R Lakhtakia R Mahapatra AK Sarkar C . Ependymoma with extensive lipidization mimicking adipose tissue: a report of five cases. Pathol Oncol Res. (2000) 6:136–40. doi: 10.1007/BF03032364, PMID: 10936790

[14] Salazar MF Tena-Suck ML Ortiz-Plata A Salinas-Lara C Rembao-Bojórquez D . Lipomatous/extensively vacuolated ependymoma with signet-ring cell-like appearance: analysis of a case with extensive literature review. Case Rep Pathology. (2017) 2017:8617050. doi: 10.1155/2017/8617050, PMID: 28286687 PMC5329680

[15] Zhao XY Yu JH Wang YH Liu YX Xu L Fu L . Lipomatous ependymoma with ZFTA: RELA fusion-positive: A case report. World J Clin Cases. (2025) 13:99746. doi: 10.12998/wjcc.v13.i1.99746, PMID: 39764539 PMC11577515

[16] Rascón-Ramírez FJ Salazar-Asencio OA Trondin A Vargas-Jiménez AC Subhi-Issa I Brin-Reyes JR . Lipomatous ependymomas of the posterior fossa. An infrequent and little-known subtype. A case report and review of the literature. Rev Espanola Patologia: Publicacion Oficial la Sociedad Espanola Anatomia Patologica y la Sociedad Espanola Citologia. (2019) 55:207–11. doi: 10.1016/j.patol.2019.08.003, PMID: 35779889

[17] Yuh EL Barkovich AJ Gupta N . Imaging of ependymomas: MRI and CT. Childs Nerv Syst. (2009) 25:1203–13. doi: 10.1007/s00381-009-0878-7, PMID: 19360419 PMC2744772

[18] Mangalore S Aryan S Prasad C Santosh V . Imaging characteristics of supratentorial ependymomas: Study on a large single institutional cohort with histopathological correlation. Asian J Neurosurg. (2015) 10:276–81. doi: 10.4103/1793-5482.162702, PMID: 26425155 PMC4558802

[19] Ertan Y Sarsık B Özgiray E Kitis Ö Dalbastı T Akalın T . Pigmented ependymoma with signet-ring cells and Rosenthal fibers: A rare variant of ependymoma. Neuropathology. (2010) 30:71–5. doi: 10.1111/j.1440-1789.2009.01031.x, PMID: 19508348

[20] Mack SC Witt H Piro RM Gu L Zuyderduyn S Stutz AM . Epigenomic alterations define lethal CIMP-positive ependymomas of infancy. Nature. (2014) 506:445–50. doi: 10.1038/nature13108, PMID: 24553142 PMC4174313

[21] Smirniotopoulos JG Chiechi MV . Teratomas, dermoids, and epidermoids of the head and neck. Radiographics. (1995) 15:1437–55. doi: 10.1148/radiographics.15.6.8577967, PMID: 8577967

[22] Vangrinsven G Bernaerts A Deckers F van Dinther J Zarowski A De Foer B . Beyond the otoscope: an imaging review of congenital cholesteatoma. Insights Imaging. (2024) 15:194. doi: 10.1186/s13244-024-01761-1, PMID: 39112725 PMC11306902

